# Phage Display on the Base of Filamentous Bacteriophages: Application for Recombinant Antibodies Selection

**Published:** 2009-10

**Authors:** N.V. Tikunova, V.V. Morozova

**Affiliations:** 1 Institute of Chemical Biology and Fundamental Medicine, Siberian Branch, Russian Academy of Science

## Abstract

The display of peptides and proteins on the surface of filamentous bacteriophage is a powerful methodology for selection of peptides and protein domains, including antibodies. An advantage of this methodology is the direct physical link between the phenotype and the genotype, as an analyzed polypeptide and its encoding DNA fragment exist in one phage particle. Development of phage display antibody libraries provides repertoires of phage particles exposing antibody fragments of great diversity. The biopanning procedure facilitates selection of antibodies with high affinity and specificity for almost any target. This review is an introduction to phage display methodology. It presents recombinant antibodies display in more details:, construction of phage libraries of antibody fragments and different strategies for the biopanning procedure.

## INTRODUCTION

In the mid-eighties, a novel molecular-biological methodology which revolutionized the engineering of peptides and proteins was developed. This approach is known as phage display. It is based on the experiments of George Smith performed in the mid-80s [1]. Initially, Smith demonstrated that an exogenous protein can be expressed on the surface of the filamentous M13 phage. This was achieved by inserting the gene that encoded a part of the EcoRI endonuclease into the ORF of the phage’s minor capsid protein pIII. Using polyclonal antibodies specific to the EcoRI endonuclease, Smith demonstrated the ability of phages carrying the chimeric EcoRI-pIII protein to specifically bind the appropriate antibodies. Furthermore, it was shown that phages with this insertion could be selected from a mixture containing wild-type phages by affine enrichment using polyclonal antibodies against the EcoRI endonuclease.

These experiments led to two important conclusions: first, using DNA-recombination methods, it is possible to create phage populations of different representativity (10^6^ - 10^11^ variants), wherein each individual phage displays a random peptide on its surface. Such populations were named "combinatorial phage libraries." Second, physical link between the analyzed polypeptide and the gene encoding it in the same phage particle provides the opportunity for easy selection of the needed variants and their identification.

G. Smith termed the result of expression of exogenous oligo- and polypeptides on the surface of viable filamentous phages "phage display." Furthermore, a method of affinity enreichment named "biopanning" was developed. According to this method, phages bearing inserted sequences with affinity to specific ligands can be selected from a phage library. The term "biopanning" was suggested in 1988 [2].

The small number of pIII molecules in the phage particle (5 copies) limits the use of phage displays in selection of synthetic immunogens. Still, attempts to obtain phages exposiung exogenous peptides as portions of the pVIII protein, which is present in 3,000 copies in each virion, were unsuccessful. Only the studies performed by Russian researchers managed to map a site on the N-terminus of pVIII that was exposed on the surface and was immunogenic but did not lead to significant disturbance of the filamentous phages’ morphogenesis [3, 4]. 

In the 1990s, phage display was used in order to expose the antigen binding fragments of immunoglobulins on the surface of the fd phage [5]. This led to a novel combinatorial approach in the development of recombinant antibodies, which was an alternative to the traditional hybridoma technology. According to this approach, the phage system allows to replace all the stages after immunization of animals and spleen removal by simple manipulations with DNA and bacteria. In addition, it reduces the time needed to obtain stable antibody-producing clones from months to weeks. It also reduces the cost of the whole procedure.

Years of using phage display have led to several important areas of application:

*Phage Display of Peptides*

- The study of receptors and mapping of antibody binding sites

- The creation of immunogens and nanovaccines

- Mapping of substrate binding sites for proteases and kinases

*Phage Display of Proteins and Protein domains*

- Selection of antibodies with specific properties

- Study of protein-ligand interactions

- Screening of expressed cDNA fragments

- Directed evolution of proteins

This review decribes the main principles and methods applied in phage display technology based on filamentous phages. Special attention is paid to the display of recombinant antibodies.

## MORPHOLOGY AND LIFE CYCLE OF FILAMENTOUS BACTERIOPHAGES 

Phage display methodology and its success are defined by the features of filamentous phages. Currently, several filamentous bacteriophages are known to infect gram-negative bacteria. The best characterized are the M13, f1, and fd phages, which infect *Escherichia coli* strains that carry an F-conjugative plasmid. The genomes of these phages have been sequenced and are 98 % homologous [6, 7]. Based on this homology and also on the dependence of infection on the presence of an F-plasmid, these phages are all termed Ff-phages.

An Ff-phage genome is a single-stranded covalently closed DNA, 6407(8) nucleotides in length, which encodes 11 genes. These genes are grouped in the genome according to their functions: the first group (genes II, V, X) encodes proteins needed for the replication of the phage DNA; the second group (genes III, VI, VII, VIII, IX) encodes surface-envelope proteins; and the third group (genes I, IV, XI) encodes proteins necessary for virion assembly. In addition, the phage DNA carries an intergenic region which contains an ori (origin of replication) site for synthesizing (+) and (-) DNA chains, as well as a site called the "packaging signal," which initiates virion assembly.

**Fig. 1:**
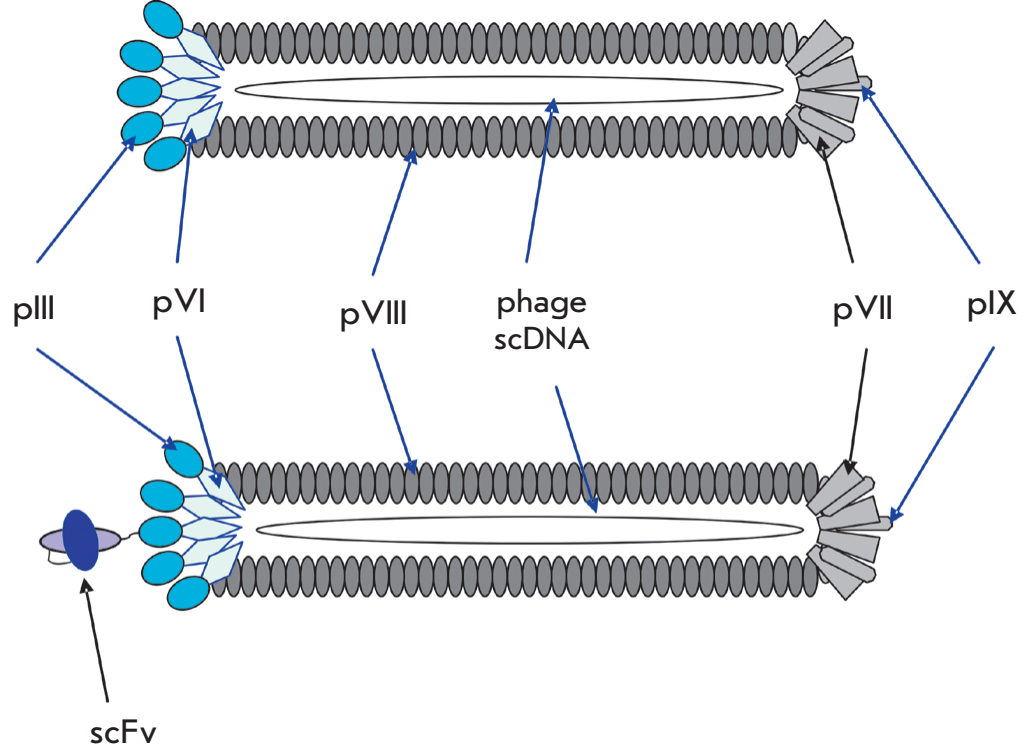
A schematic representation of a filamentous phage: (a) wild-type filamentous phage; (b) filamentous phage-based phage antibody

Ff-phage DNA is enclosed in a flexible cylinder comprised of approximately 2,700 molecules of pVIII (Fig.1). One end of the Ff-phage carries 5 copies of the minor surface proteins pIII and pVI; the other, pVII and pIX.

Infection of *E.coli* cells by the Ff-phage starts with a specific interaction between pIII and the top of the F-pilus, which is a protein tube made up of pilin subunits. Retraction of the pilus, caused by de-polymerization of pilin subunits, pulls the bacteriophage towards the cell [8]. After the phage DNA penetrates into the cytoplasm, it is turned into a replicative plasmid-like molecule (RF molecule) by the *E.coli* replication enzymes. This molecule then acts as a template for transcription and translation of phage proteins.

Phage protein production is enhanced as the number of RF-molecules increases, and after reaching a certain concentration of the pV protein, the newly synthesized ssDNA forms a DNA-pV complex, which is used in bacteriophage assembly. Assembly takes place in an area of tight contact between the cell wall and the cellular membrane and continues until the end of the phage DNA is freed and the phage leaves the cell. Assembly of the virions does not lead to cell lysis, and the infected cells continue to divide, although slower than uninfected cells [9].

## FILAMENTOUS PHAGE-BASED VECTOR SYSTEMS

A number of novel vector molecules have been constructed on the base of filamentous phage DNA. These so-called "phagemids" combine the characteristics of plasmids and phages [10]. Phagemids contain a replication origin and the packaging signal of the Ff-phage, as well as a replication origin for the chosen plasmid, gene III, a polylinker, and an antibiotic-resistance gene [11].

**Fig. 2:**
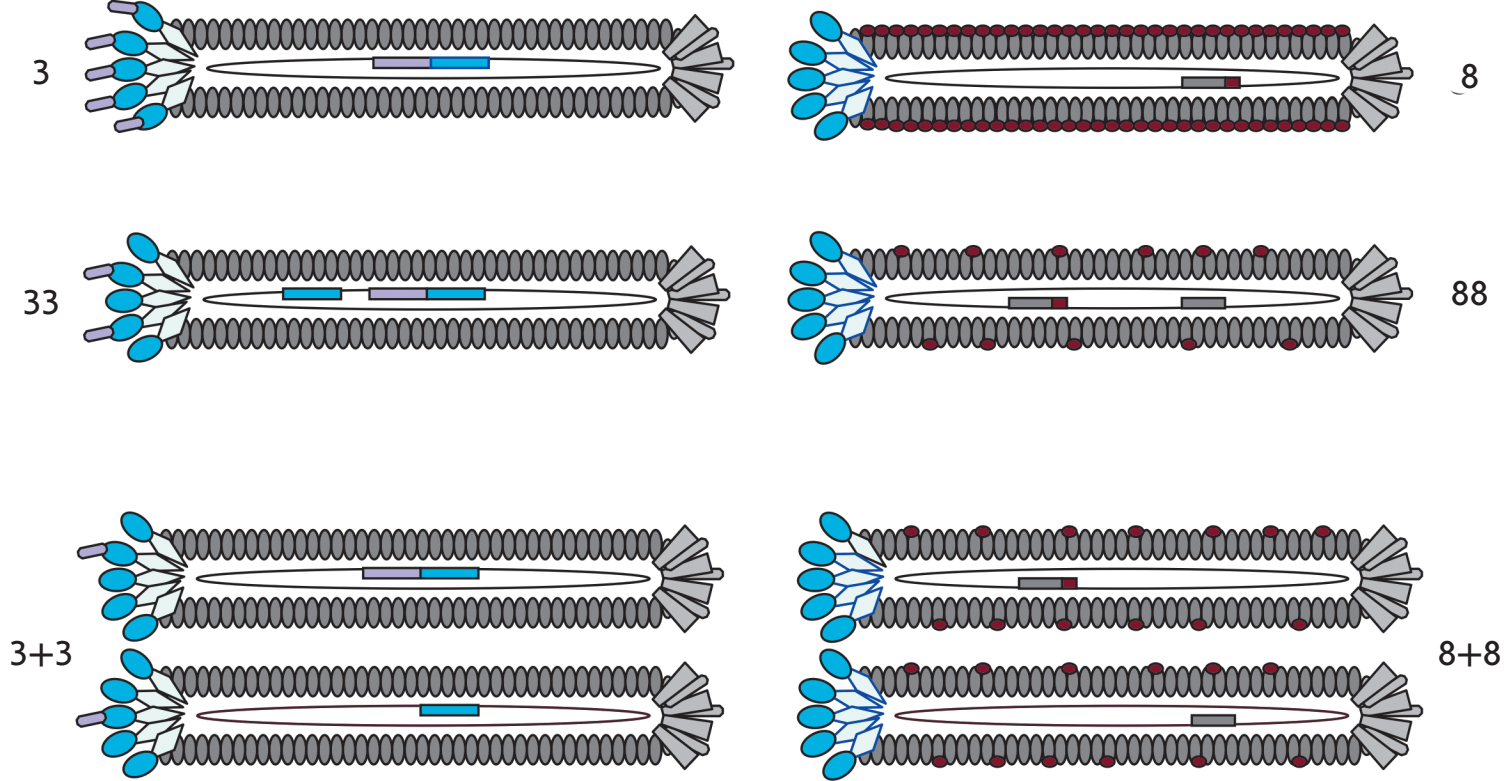
Types of vector systems for peptide and protein display based on filamentous phages. Lilac and red represent the exogenous inserts into the genes encoding the pIII and PVIII proteins, respectively

Several phage display systems were developed on the base of five capsid proteins [12], but systems based on the minor envelope protein pIII or the major envelope protein pVIII are mainly used (Fig.2). These vector systems are termed system 3 and 8, respectively [10].

Depending on the size of the exogenous insert, the envelope protein of the phage may lose its normal virion assembly or cell infection, both of which lead to decreased viability of the phage. In order to restore infectivity of phages with inserts, specialized vector systems were developed using introduction of an additional gene encoding wild-type pIII or pVIII protein. The vector systems, termed 33 or 88, carry both the wild-type phage gene and the recombinant gene. The vector systems referred to as 3+3 and 8+8 have the recombinant gene on a phagemid, while the additional wild-type gene is introduced into the *E.coli* cells via a "helper" phage. In both cases, the replicated phages carry both normal and hybrid proteins and can replicate, despite the presence of exogenous inserts.

The 8+8 vector system exhibits multivalence: both wild-type and recombinant pVIII proteins are exposed on the surface of the phage particle, but since pVIII is a major envelope protein, only several hundred exogenous fragments can be exposed. Such a high valence is useful when low-affinity ligands need to be selected.

On the other hand, the main characteristic of the 3+3 vector system is its virtual monovalence: both recombinant and wild-type pIII proteins are exposed on the surface of the phage particle, and the number of recombinant protein copies varies from 0 to 5 for each single virion. Notably, only 10 % of the phages carry even one copy of the chimeric protein, and the percentage of phages carrying 2 or more molecules of recombinant pIII is considerably smaller. About 90 % of the phages carry no chimeric protein [13]. This low valence leads to limited avidity, which in turn allows the selection of high-affinity molecules, and the 3+3 system is mostly used for selection of antibody fragments.

## MAIN STEPS OF A PHAGE DISPLAY PROTOCOL

In order to select for target antibodies with the desired characteristics, two important steps are required: first, an adequate phage antibody library should be used; and second, the right biopanning strategy should be chosen.

**Fig. 3:**
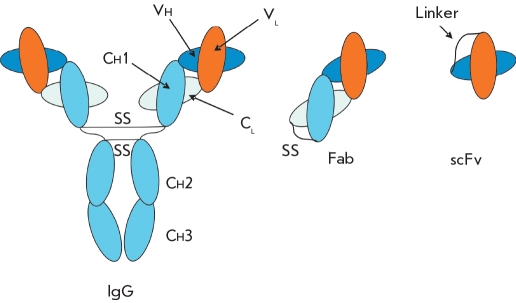
Natural immunoglobulin G molecule and the antigen-binding fragments of the immunoglobulin

**Fig. 4:**
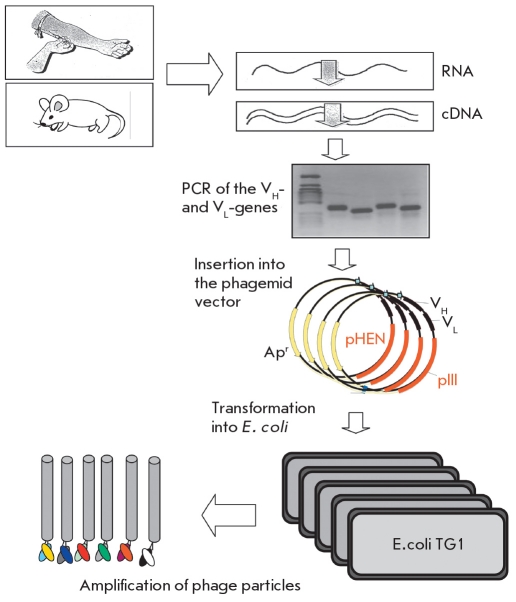
A schematic representation of the construction of an antibody fragment phage library

A combinatorial phage library of antibodies is a phage population in which each individual phage exposes a unique antigen-binding antibody domain on its surface as a part of a chimeric pIII protein. This antibody domain is most often a single chain-variable fragment (scFv) or Fab-fragment (Fig.3). The structural and functional traits of these antibody fragments are reviewed in [14]. The creation of such a library involves the cloning of PCR-amplified DNA fragments encoding Fab or scFv from different sources into the ORF of the pIII protein (Fig.4). This yields a repertoire of phagemids, and each phagemid includes a DNA fragment encoding an individual antigen binding domain. Transfection of *E.coli* cells with this phagemid repertoire provides a library of phages, and each phage exposes an individual combination of variable heavy- and light-chain domains on its surface. DNA ligation and bacterial transformation are the key steps, since they determine the size of the library [15].

Such libraries are characterized by the affinity level of the obtained antibodies, as well as the size of the library and its functional size. The affinity of the selected antibodies is primarily determined by the size of the library that is limited by the efficiency of *E.coli* transformation. The size of the library is the number of clones growing after transformation of *E.coli* with the whole phagemid population. A much more important factor is the functional size of the library, which is the number of clones that carry correctly assembled genes without deletions, frame-shifts, or improper stop-codons. The functional size of the library is always smaller than the initial size, but it is the key parameter that determines the properties of the antibodies to be selected [16].

**Fig. 5:**
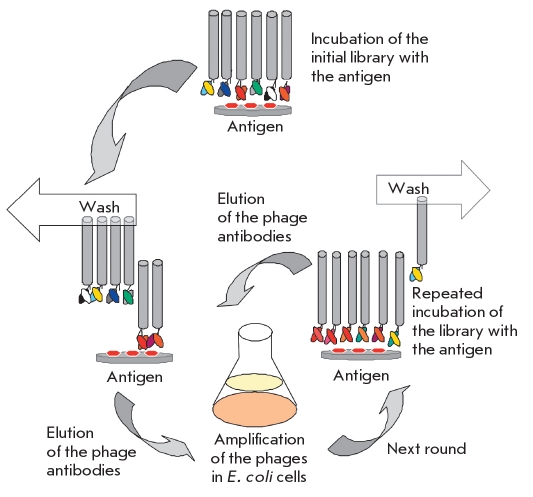
A schematic representation of the biopanning procedure

Selection of the target antibodies requires a biopanning procedure, an affine enrichment of the library by antibodies specific to the desired antigen (Fig.5). This involves the incubation of an immobilized antigen with the phage library. The unbound phage antibodies are removed, and the bound ones are eluted and used for infecting *E.coli*, where they replicate. These phages are then extracted and used for the next round of biopanning. Ideally, one round of biopanning should be enough, but nonspecific binding limits the enrichment during each single round; so, several rounds of selection are usually required. Then, individual antibodies directed against a specific antigen are selected from the enriched library.

**Types of phage antibody libraries**

There are two types of *in vitro-*made phage antibody libraries: the so-called "natural" and "synthetic" libraries, which are further subdivided according to the used gene repertoire.

Currently, a number of phage display antibody libraries have been fully or partially obtained from natural sources; thus mRNA was extracted from peripheral lymphocytes, bone marrow or the spleen [17, 18]. Libraries of human antibodies are of special interest, since the selected antibodies can be used for the development of therapeutics. Since most of the families encoding human V-genes are rarely used in the immune response, only the often-occurring gene families are usually used for library construction: V_H1_ - V_H3_ (from the heavy-chain gene family) and V_K1_ - V_K4_, and also V_≤1_ - V_≤3_ (from the light-chain gene family). Both random recombination of the light- and heavy-antibody chains and the variability of the hyper-variable regions (complementarity determining region - CDR) of the V_H_ and V_L_ chains play an important role in the variability of the library.

Natural antibody libraries are divided into "immune" and "naive" libraries. Immune libraries based on peripheral lymphocytes extracted from humans immunized by a certain antigen are valuable for medical research, since they provide increasing probability of selecting antibodies that could be used for therapy [19].

"Naive" libraries based on lymphocyte mRNA extracted from unimmunized healthy people are used for obtaining antibodies directed against a wide multitude of antigens, including autoantigens [20]. These "naive" libraries mostly represent the germline diversity of antibodies.

Synthetic libraries were introduced in order to increase the variety and size of the library and to enhance the antibody’s characteristics. Synthetic phage antibody libraries are divided into two groups:

1) Synthetic libraries based on a single-core V-gene;

2) Synthetic libraries based on a multiple-core V-genes.

Libraries of the first type are based on a single gene, which is then mutagenized in all the CDRs or just the CDRs in the V_H_-domains. In this case, library diversity is limited by the degeneracy of the synthetic DNA which is used to change the sequence of the CDR-loops.

Synthetic libraries based on a single-core sequence present several advantages. They are easier to construct, and the obtained antibodies can be analyzed in less time. But the issue remains as to whether a single core sequence allows the correct folding of various antigen-binding CDRs providing a wide range of high-affinity antibodies.

Synthetic libraries of the second type are based on tens of genes, which are mutated in all the CDRs or in the CDRs in the variable domains of the heavy chains.

Different types of genes (Fab and scFv) can be used for creating both natural and synthetic phage libraries. Thus, combinatorial phage libraries of Fab- or scFv- antibody fragments can be obtained.

After MacCafferty and his collaborators demonstrated the possibility of creating a scFv library on the surface of a filamentous phage [5] in 1990, much research has been aimed at developing such libraries and studying the selected recombinant antibodies. In order to create a scFv library, populations of V_H_- and V_L_-genes are combined into a single DNA sequence by using an oligonucleotide encoding a flexible hydrophilic peptide (Fig.3). This linker consists of glycine and serine residues (Gly_4_ Ser)_3_ which make it flexible and resistant to proteases. Then, the obtained scFv-genes are cloned into an appropriate vector (pHEN1, pHEN2, pSEX, etc.), which expresses the scFv-antibodies as part of the chimeric pIII protein. The heavy chain is most often immediately downstream from the leading sequence, while the light chain is fused to the N-terminal pIII sequence. The peptide linker facilitates the association between the V_H_- and V_L_ domains necessary for forming the antigen binding surface, so disulfide bonds between the chains are not needed.

For further purification and characterization, it is convenient to produce the scFvs as secreted proteins. If pHEN or pSEX vectors are used, an amber stop codon inserted between the 3’-terminus of the scFv gene and the 5’-terminus of the pIII gene provides production of scFv. in nonsuppressor *E. coli* strains transformed by the phagemids [21].

Fab-fragments are heterodimers consisting of a light immunoglobulin chain (V_L_-C_L_) combined with a variable domain and the first constant domain of a heavy chain (V_H_-C_H1_). The chains interact after both have been synthesized, and this interaction stabilizes the formation of the antigen-binding site (Fig.3). Creating Fab-libraries involves cloning a light chain and an Fd-domain (V_H_ + C_H1_) into the appropriate vector. The gene encoding the Fd-domain is combined with the region encoding the C-terminus of the pIII phage protein. Thus, the Vl-gene and the Vh-gene, which is fused with the pIII phage protein, are transcribed as a polycistronic mRNA under the control of the chosen *E.coli* promoter*.* The N-terminus of each polypeptide consists of a leading sequence, which directs it towards the cellular membrane where virion assembly takes place.

A wider range of Fab-fragments can be obtained by transforming cells with plasmid bearing V_H_C_H1_-chains and then transfecting the same cells with a phage population exposing V_L_C_L_-chains. In this case, complete chain recombination must be assured, so that the chains are all present in the phage particle for the following selection procedure. In order to achieve the recombination of the H- and L-chains *in vivo*, both the plasmid and the phagemid contain loxP-sites for the phage recombinase P1, and the *E.coli* strain is modified to produce phage recombinase [22].

Fab-fragments are usually more stable after purification as compared to scFvs. They also have a longer elimination half-life and thus better pharmacokinetic and pharmacodynamic qualities [14]. In addition, Fab-fragments can be turned into full-sized immunoglobulins relatively easily by combining the C-terminus of the C_H1_ domain with the Fc-fragment.

## LIBRARY CONSTRUCTION

Different sources of gene repertoire are used for the construction of phage display antibody libraries, depending on the library type. Naive and immune libraries are constructed using naturally reorganized genes, which encode the variable immunoglobulin domains of healthy donors or donors immunized with a certain antigen, respectively (Fig.4). mRNA from the antibody-producing lymphoid cell line is isolated for this purpose: peripheral blood lymphocytes are mainly used, but splenocytes [23, 24], tonsil cells, and B-lymphocytes from the bone marrow, have been used as well [25, 26]. A preliminary *in vitro* immunization of peripheral lymphocytes can also be used to construct an immune antibody library [27, 28].

cDNA is then synthesized on the base of isolated mRNA, and both oligo-dT and statistically devised hexanucleotides and specific primers can be used that yield cDNA copies of all the possible variable domain genes [29]. Also, one or several primers can be used to limit the range of amplified genes to one or several variable domain gene families or antibody isotypes [30]. Information from the DataBase Kabat [31] or V BASE can be used for primer development. The primer sequence usually includes restriction sites for cloning the PCR-products into the appropriate vectors.

**Naive library construction**. Phage display library of human scFv was constructed by Marks and his colleagues [30]. Peripheral blood lymphocytes isolated from two volunteers were used as a gene source. The primers used for cDNA synthesis were complementary to the conservative regions of the genes encoding the ≤- and ≤-type light chains and the IgM and IgG heavy chains. The cDNAs were than cloned into the pHEN1 vector, yielding two libraries: V_H≤_-V_L_ (2.9 · 10^7^ clones) and V_H≤_-V_L_ (1.6 · 10^8^ clones) [30]. Various protein-, hapten- [30], and autoantigen-specific antibodies were selected from these libraries [30, 32], although the affinity constants of the selected scFv’s did not exceed 10^7^ M^-1^. Still, high-affinity antibodies can be extracted from relatively small-sized libraries. For instance, a scFv-library containing 4 · 10^7^ clones yielded scFvs specific to steroid hormones, and their affinity constants were 10^7^ - 10^8^ M^-1^ [33]. Single-chain antibodies specific to the tumor necrosis factor (K_a_ = 10^7^ - 10^8^ M^-1^) were selected from a naive library of 2 · 10^8^ of independent clones. In order to increase the representativity of this library, the protocol for obtaining scFv-genes was modified. In contrast to previous work [30], in which the scFv-genes were constructed by 3-component PCR (V_H_ - linker - V_L_), a more efficient two-component reaction was used, involving a restriction site in the linker sequence [16].

Further experimental efforts were aimed at constructing the wider variety of antibody fragments, since antibodies with nanomolar affinity to any antigen can be selected from a large repertoire (10^10^-10^11^). Such a library (1.4 · 10^10^ clones) was developed on the base of the genetic material extracted from the lymphoid cells (peripheral blood lymphocytes, tonsil cells, and bone marrow B-lymphocytes) of 43 unimmunized donors [25]. V_H_- and V_L_-gene repertoires were preliminarily cloned into pCantab 6 and pCantab 3His_6_ vectors, respectively. The affinity of the antibodies obtained from this scFv library exceeded 10^9^ M^-1^. It was known that antibodies with such affinities usually appeared after a secondary immune response. This library also yielded antibodies specific to cell surface antigens [34] and an antibody that could specifically bind carbohydrates [35]. Also, antibodies specific to various toxic agents were obtained from this library. This result demonstrated an advantage for large libraries when compared to the hybridoma technique, because toxic agents were impossible to use for immunization [10]. Naive scFv libraries were also used to obtain scFv specific to neurotoxins [36], to apoptosis proteins [37], etc.

The creation of a large naive library of Fab fragments involved a two-step cloning strategy [24]. First, amplified V_H_-, V_L≤_- and V_L≤_-genes were cloned into vectors bearing the C_H1_, C_L≤_- and C_L≤_-genes, respectively. After that, restriction fragments which were obtained from the initial V_H_C_H1_, V_L≤_C_L≤_, and V_L≤_C_L≤_ gene repertoires were used instead of the usual PCR products. This approach made the cloning procedure more effective. Similarly to the creation of scFv libraries, the oligonucleotides for the amplification of the variable domains were chosen to maximize the output of the gene repertoire. The resulting Fab-fragment library contained 3.7 · 10^10^ independent clones, and the affinity of the antibodies selected for a wide range of antigens varied from 2.7 · 10^7^ to 3.7 · 10^8^ M^-1^.

**Synthetic library construction.** Combinatorial libraries are characterized not only by size, which is determined by the number of clones, but also by representativity, which is the overall number of different V_H_-V_L_ combinations. One way to extend the representativity of a library is to increase the number of donors, and this way is particularly used for natural libraries. However, the antibody-encoding gene repertoire still does not exceed the variability of the lymphoid cell genes available for library construction. The potential antibody repertoire in the human organism is supposed to reach 10^12^, but the antibody-producing cells represent only a portion of that amount at a moment [37, 38]. A second way to extend the representativity of a library is to replace natural CDRs with synthetic ones. In this case, it is possible to construct a combinatorial library including all the possible antigen-binding sequences of antibodies. Such phage display libraries are usually termed semi-synthetic or synthetic, depending on whether one or both variable domains contain synthetic CDRs. An ideal way to produce a repertoire that would encompass all the possible antibody sequences would be to chemically synthesize all 6 randomized CDRs and then connect them via various structural domains. But usually CDR3 of the heavy chain is randomized, since it is the most significant component of the antigen binding site [39].

The first semi-synthetic library was based on one heavy chain in which CDR3 was replaced with 10^20^ different sequences and one intact light chain [37]. This library contained 5 · 10^7^ clones, and the affinity of selected antibodies varied from 10^7^ to 10^8^ M^-1^.

Later, single-core semi-synthetic libraries were constructed using mutagenesis to change all CDRs in the V_H_-domain [40]. The positions for mutagenesis were chosen according to the special variability of natural antibody sequences. These positions were randomized using the codons which are mostly found in natural antibodies that provided good solubility of selected antibodies.

Besides libraries based on a single-core sequence, some libraries based on a repertoire of V_H_-genes have been constructed [41, 42]. Thus, the Nissim library was based on 50 V_H_-genes encoding most of the human V-segments and random nucleotide sequences encoding the V_H_ CDR3. These sequences varied from 12 to 36 b.p.. This approach yielded 9 V_H_-gene repertoires whith different lengths of V_H_CDR3 and, thus, 9 phagemid libraries. The size of the overall library was 10^8^ independent clones, and this library yielded antibodies specific to a whole range of antigens [42].

Another commonly used library is the Griffin 1 library. It was constructed in a similar way to the previous library, based on 50 germline V_H_-genes. In addition, 6 V_L_-genes, corresponding to the 6 main subtypes of ≤- and ≤-chains, were used. The library contained 10^8^ clones, and it yielded antibodies specific to the soluble CD4 receptor [43], human interleukin 6 [44], orthopoxviruses [45], and the Ebola virus nucleoprotein [46]. This library was also used to select catalytic antibodies [47] and other ones [48].

The affinity of the selected antibodies yielded by these semi-synthetic and synthetic libraries was not high due to several factors. It is known that V_H_ CDR3 is highly variable in length in natural antibodies and can consist of up to 24 amino acid residues. Creation of synthetic CDR3 sequences with varying lengths and completely random structures is inefficient. Moreover, the structural conformation of antibodies with synthetic CDRs could be incorrect (compared to natural conformations) precisely at the CDR loops [49]. In order to avoid this difficulty, limited randomization and introduction of flanking structures that enclose the completely random amino acid sequences were used [49]. The resulting library contained 3.6 · 10^8^ clones, and scFv’s specific to different antigens were selected from it, although the affinity of the selected antibodies was still low (4 · 10^5^ - 10^7^ M^-1^).

Further steps for avoiding the above-mentioned limitations depend on the use of so-called "master-genes," which are the genes encoding variable domains with specific frame regions flanking the various randomized CDRs. The HuCAL library was constructed on the base of V_H_- and V_L_-gene families, which are mostly used in the immune response. Hence, 7 V_H_-genes and 7 V_L_-genes yielded a basic collection of 49 different scFv genes. All the genes were synthesized to avoid the codons for amino acid residues that promote aggregation [50]. This library was constructed according to the position of key amino acid residues in CDRs and the frame regions, the length of the CDRs, and the level of their variability. The synthesis of oligonucleotides encoding CDR3 involved the use of cassette trinucleotide mutagenesis, which removed the TAG termination codon and uncommonly used codons. This library contained 2 · 10^9^ clones, and antibody fragments were selected from this library for a wide range of antigens, including peptides, proteins, and whole cells. The affinity constants were near those of a secondary immune response (10^9^ M^-1^). 3D structures have been created for all the used consensus structures. That has allowed further study of the source for the variability of natural structural motifs of human antibodies, and also the correlations between antibody structure, its affinity, specificity, and the bound antigen class [50, 51].

The n-CoDeR™ library was constructed in a similar way [52]. A single master gene modified by introduction of different *in vivo*-formed CDRs have been used. Since the genes contained natural CDRs, an encreased amount of correctly assembled and functional molecules was guaranteed. Moreover, computer analysis showed that the antibodies produced from the CoDeR™ library were less immunogenic. This library yielded antibodies that were specific to carbohydrates and human autoantigens, with an affinity exceeding 10^9^ M^-1^.

**Construction of immune libraries. ** Despite the fact that many universal naive synthetic and semi-synthetic libraries have been constructed in recent years, it has become clear that the main problem is not the creation of an enormous repertoire of antibody fragments, but conservation of this repertoire for extended periods of time, and keeping it without failure in antibody structures. Also, the larger the library, the more practically difficult it is to work with it. For instance, amplification of a library with 10^10^ clones takes tens of liters of culture medium. This is one of the main reasons why smaller libraries containing a narrowed repertoire of antibodies are preferable.

Immune libraries have two main features: they are enriched with antigen-specific antibodies, and the affinity of some of the antibodies has increased during the immune response. So, these libraries contain an extended amount of clones producing high-affinity antibodies specific to the antigen used for the immunization and that appeared as a result of a secondary immune response. It is assumed that immune libraries containing 10^6^ clones can yield some antibody fragments that specifically bind the antigen used for immunization. In comparison, naive libraries must contain at least 10^8^ individual clones in order to possess the original variability of antibodies [53].

The first immune libraries were constructed against HIV [54], the respiratory syncytial virus [55], hepatitis B virus [56], herpes simplex virus, and cytomegalovirus [57, 58]. Libraries have also been constructed against human autoantigens [59-61] and against antigens that cause allergic reactions [62].

Libraries based on the lymphocyte genetic material isolated from patients were constructed to select antibodies against specific tumor markers [63 - 68]. Immune libraries have also been made against the hepatitis A virus [19], chicken pox virus [69], orthopoxviruses [70], etc. Recently, virus-neutralizing scFv fragments specific to the flu virus H5N1 [26, 71] and virus-neutralizing Fab-fragments specific to the rabies virus [72] were extracted from immune libraries. Full-size human antibodies against orthopoxviruses [73, 74], neutralizing antibodies against hepatitis A [75], and neutralizing antibodies specific to the B glycoprotein of cytomegalovirus [76] have been made using antibody fragments isolated from immune libraries. 

A serious advantage of immune libraries is the possibility to select high-affinity antibodies, which appear after viral infections or cancer, and antibodies specific to autoantigens, which are present in patients with autoimmune diseases. Analysis of these antibodies can help in the identification of the antigen epitopes responsible for the humoral immune response. Another advantage of immune libraries is that weakly immunogenic antigens can be used for antibody selection.

## AFFINITY SELECTION OF ANTIBODIES FROM LIBRARIES

The next important step after constructing a library or choosing an available one is the enrichment of the original antibody repertoire with the antibodies specific to a target antigen (Fig.5). This procedure is called "biopanning" or affinity enrichment. Below, we present several biopanning strategies.

**Biopanning with immobilized antigens.** Traditionally, biopanning has been performed using an antigen absorbed on a plastic surface, for instance immunotubes (Maxisorb tubes; Nalge Nunc Intl., Naperville, IL) or enzyme immunoassay plates [30, 78]. Biopanning can also be performed using affinity chromatography, which involves the immobilization of the target antigen in a column [43, 79]. The column is then washed to remove unspecific antibodies, and the bound specific phage antibodies are eluted from the column and amplified in *E.coli* cells*.* BIAcore chip sensors can also be used as an antigen-binding medium for affinity selection [80]. Using this method for biopanning, it is important to keep the conformational stability of the antigen. Some phage antibodies selected against adsorbed antigens cannot bind the antigen in its native conformation. One method to avoid this problem is to use indirect antigen binding by incorporating antigen-specific antibodies [81].

Elution of the specifically bound antibodies is performed by using acid solutions, such as HCl or a glycine buffer [78, 82], or basic (alkaline) solutions, such as triethylamine [30]. It is very important to neutralize the phage antibody eluate right after elution, adjusting the pH to 7.2-7.4. Antibodies can also be eluted by cleaving a specially introduced site between the antibody and the pIII protein with a protease [83]. Moreover, antibodies can also be eluted by adding an excess of antigen, since this antigen will compete with the immobilized one binding the antibodies [16, 43].

**Biopanning using antigen solution.** Immobilizing the antigen on a solid surface often causes conformational changes of the antigen molecules. To avoid conformational changes, a set of methods are used to bind antigens to antibodies in a solution. The use of tagged soluble antigens allows precise quantification of the antigen concentration [84], and thus the use of minimal concentrations, which leads to selection of high affinity antibodies. After incubating the antibodies with an antigen conjugated with biotin, phages bound to the tagged antigen are collected with avidin- or streptavidin-covered paramagnetic beads. Then, the specifically bound phages can be eluted from the antigen and characterized. A drawback of this method is the co-selection of anti-streptavidin antibodies. This problem can be solved by adding one more step; incubation of the antibodies with streptavidin-covered beads so as to remove any streptavidin-specific antibodies.

**Biopanning on cells.** Direct antibody selection against cell surface markers can be performed using either a cell monolayer or a cell suspension. The unbound phage antibodies are removed by washing the culture dishes in the case of a monolayer and by centrifugation in the case of a suspension. In order to optimize the selection of antigen-specific antibodies and minimize the selection of nonspecific antibodies, a negative selection procedure can be performed before, after or simultaneously with the positive selection [49]. Simultaneous positive and negative selection creates competitive conditions: a small number of antigen-positive (target) cells and an excess of antigen-negative adsorbing cells, which bind the unspecific antibodies from the phage library. In order to collect the cells with the bound phage antibodies, fluorescently labeled antibodies specific to another antigen on the target cell surface are added to the suspension. The cells are then collected by a FACS-sorter.

Using a similar method, anti-Rh(D) Fab-antibodies, which are very promising for clinical use, have been extracted from a Fab-fragment library [85]. Similar approaches can be used for identifying tumor-specific antigens and also as a highly productive protocol for selecting antibody fragments specific to conformation-dependent cell surface markers. A scFv-library was subjected to 3 rounds of positive selection using human melanoma cells and negative selection using human peripheral blood mononuclear cells [64]. Two scFv, which could bind melanoma cells according to the enzyme immunoassay and FACS, were selected with this procedure [64]. Similar selection can be performed using fragments of various tissues.

A novel method has been developed for selecting antibodies which can penetrate eukaryotic cells via receptor-dependent endocytosis. In this case, selection is performed under conditions that stimulate active endocytosis. Thus, after lysis of the used cells, a population of phage antibodies which are able to penetrate eukaryotic cells can be obtained [86, 87]. It is suggested that such antibodies can act as vehicles for introducing various drugs into cells.

*In vivo*** biopanning.** This method involves a direct injection of the antibody repertoire into an animal’s organism. Tissues are then removed and phage antibodies bound to tissue-specific cell markers can be extracted. A similar approach was used for peptide libraries [88]. I*n vivo* biopanning has several advantages: first, the extracted antibodies specifically bind intact cell targets; second, antibodies binding nontarget cell surface proteins and blood plasma are immediately eliminated. Antibodies obtained via *in vivo* biopanning or cell biopanning can be very useful for the functional analysis of newly discovered receptors and in the search for potential targets of novel drugs.

Notably, most of the existing selection procedures involve only one or several antigens. However, there are biopanning strategies that allow parallel selection for antibodies against a population of antigens; the so-called "2D display." Thus, it has been proposed to incorporate multiplex flow cytometry for high-output simultaneous selection of individual antibodies against a wide range of antigens present on the surface of cells [89]. An alternative method of selection against a population of antigens is the combinatorial selection of antigen-antibody pairs which are able to replicate. Such an approach was used for selecting a scFv phage library for antibodies specific to antigens from a yeast library [90].

## PROBLEMS AND SUCCESSES OF DISPLAYS BASED ON FILAMENTOUS PHAGES

Many researchers have faced a number of problems while constructing and using libraries based on filamentous phages. For instance, the low concentration of the original antigen-specific lymphocytes in the population can be a problem while constructing immune libraries. This and similar problems can be avoided by preliminary enrichment of the B-lymphocyte population, using antigen molecules conjugated with magnetic beads [91, 92].

Another difficulty is selective loss of high-affinity antibodies during biopanning cycles. Another problem is the decrease of the share of phages with full-sized inserts during library amplification and propagation. Analysis of the antibody-encoding gene’s size showed that the number of clones with defective inserts increased after even one round of biopanning [16]. Phages with smaller inserts (usually with an absent V_H_ region) tend to grow more quickly than phages with full-sized inserts, which leads to their more effective amplification. That is why using the appropriate biopanning strategy is so important. Varying the elution and screening conditions during selection can help to select high-affinity antibodies.

Another problem is the big difference between the theoretically possible and the actual variety provided by the phagemid population. This difference cannot be fully explained by cell transformation efficiency. It can also be caused by cell toxicity of the antibodies, incorrect protein folding or assembly, competition between the chimeric and wild-type pIII proteins, proteolysis of the antigen-binding site on the surface of the phage, and several other factors.

Nevertheless, despite the existing methodological problems, phage display techniques are showing considerable progress. This technology is a very promising instrument for several reasons. Human antibody fragments selected from phage display libraries could be useful in the construction of fully human antibodies that have lower immunogenicity than chimeric or humanized antibodies and would be more valuable for therapeutic use. Antibody fragment libraries allow the rapid isolation of specific antigen-binding domains without any of the limitations caused by the availability of donor lymphocytes or cell-fusion difficulties. Another advantage of phage display libraries is that one library can be used for multiple screening against different antigens. One more advantage of this technology is the absence of laboratory animal use, the relative simplicity of the protocols, and the possibility of screening a large amount of candidate molecules in a short period of time. A very important feature is the possibility of selecting antibodies against a wide range of antigens, including toxic substances or highly dangerous viruses, which cannot be used for immunization because of ethical reasons. Phage display technology has proved its effectiveness in the development of therapeutic antibodies. In recent years, more than 14 antibodies on the U.S. pharmaceutical market have been obtained using phage display methods [15]. All these facts explain the interest of large companies, such as Morphosys GmbH (Germany; http://www.morphosys.com); Cambridge Antibody Technology (United Kingdom; http://www.catplc.co.uk), Dyax (USA; http://www.dyax.com) and others, in the development and application of antibody fragment phage display libraries.

## Acknowledgements

This work was supported by RFBR grant 07-04-12100-ofi, 09-04-01546-a, 07-04-92168-NCNI_a, NATO SFPP 982833, and the "Fundamental science for medicine - 2008" program of the presidium of the Russian Academy of Sciences.
